# Dual function of active constituents from bark of *Ficus racemosa* L in wound healing

**DOI:** 10.1186/s12906-018-2089-9

**Published:** 2018-01-25

**Authors:** Nisansala Swarnamali Bopage, G. M. Kamal Bandara Gunaherath, Kithsiri Hector Jayawardena, Sushila Chandrani Wijeyaratne, Ajita Mahendra Abeysekera, Seneviratne Somaratne

**Affiliations:** 1 Department of Chemistry, The Open University of Sri Lanka, Nawala, Nugegoda, Sri Lanka; 2 Department of Zoology, The Open University of Sri Lanka, Nawala, Nugegoda, Sri Lanka; 3 Department of Botany, Sri Jayewardenepura University, Nugegoda, Sri Lanka; 4 Department of Chemistry, Sri Jayewardenepura University, Nugegoda, Sri Lanka; 5 Department of Botany, The Open University of Sri Lanka, Nawala, Nugegoda, Sri Lanka

**Keywords:** *Ficus racemosa* L., Wound healing, Scratch wound assay, Cell migration enhancement, Baby hamster kidney (BHK 21) cells, Madin-Darby canine kidney (MDCK) cells, Kirby Bauer disc diffusion assay, Lupeol, Lupeol acetate, *β*-sitosterol

## Abstract

**Background:**

Different parts including the latex of *Ficus racemosa* L. has been used as a medicine for wound healing in the Ayurveda and in the indigenous system of medicine in Sri Lanka. This plant has been evaluated for its wound healing potential using animal models. The aim of this study was to obtain an insight into the wound healing process and identify the potential wound healing active substance/s present in *F. racemosa* L. bark using scratch wound assay (SWA) as the in-vitro assay method.

**Method:**

Stem bark extracts of *F. racemosa* were evaluated using scratch wound assay (SWA) on Baby Hamster Kidney (BHK 21) and Madin-Darby Canine Kidney (MDCK) cell lines and Kirby Bauer disc diffusion assay on common bacteria and fungi for cell migration enhancing ability and antimicrobial activity respectively. Dichloromethane and hexanes extracts which showed cell migration enhancement activity on SWA were subjected to bioactivity directed fractionation using column chromatography followed by preparative thin layer chromatography to identify the compounds responsible for the cell migration enhancement activity.

**Results:**

Dichloromethane and hexanes extracts showed cell migration enhancement activity on both cell lines, while EtOAc and MeOH extracts showed antibacterial activity against *Staphylococcus* and *Bacillus* species and antifungal activity against *Saccharomyces spp.* and *Candida albicans*. Lupeol (**1**) and *β*-sitosterol (**2**) were isolated as the potential wound healing active compounds which exhibited significant cell migration enhancement activity on BHK 21 and MDCK cell lines (> 80%) in par with the positive control, asiaticoside at a concentration of 25 *μ*M. The optimum concentration of each compound required for the maximum wound healing has been determined as 30 *μ*M and 35 *μ*M for **1** and **2** respectively on both cell lines. It is also established that lupeol acetate (**3**) isolated from the hexanes extract act as a pro-drug by undergoing hydrolysis into lupeol in the vicinity of cells.

**Conclusion:**

Different chemical constituents present in stem bark of *Ficus racemosa* L show enhancement of cell migration (which corresponds to the cell proliferation) as well as antimicrobial activity. This dual action of *F. racemosa* stem bark provides scientific support for its traditional use in wound healing.

## Background

A wound is an injury that results in opening or breaking of the skin [1]. Because the skin is the protective barrier, wounds should be healed rapidly and efficiently within the shortest possible time [2]. Various complications can occur if wound healing does not set forth in an orderly and timely manner [3]. Many of the drugs currently used for wound management are expensive while some shows allergic reactions and drug resistance [4, 5]. Phytomedicines have generated much interest for the treatment of skin ailments as they are affordable and supposedly safe [6]. Hence, the possibility of deriving cost effective therapies from plant based traditional medicine has been explored [7, 8].

*Ficus racemosa* L. (Family Moraceae) is distributed widely throughout the warmer parts of Asia, Africa, America, and Australia [9]. It is commonly known as Cluster Fig in English. All parts *F. racemosa* are regarded medicinally important in Ayurveda and have been used extensively in the treatment of biliary disorders, jaundice, dysentery, diabetes, diarrhea and inflammatory conditions [9–11]. In addition, various parts of this plant and latex either in the raw form or as different preparations have been used in Ayurveda for the treatment of wounds [10–17]. Apart from the wound healing activity of the stem bark extracts of *F. racemosa* on a rat model [18], biological properties such as hepatoprotective [19], chemopreventive [20], hypoglycemic [9, 21–23], anti-inflammatory [24], anti-pyretic [23], anti-tussive [25], anti-diuretic [26], anti-cholinesterase [9], mosquito-larvicidal [27], anti-bacterial [28] and antioxidant [22] activities also have been reported. Many medicinal herbs including *F. racemosa* root have been evaluated for the wound healing properties on animal models using excision, incision and dead space models [29, 30].

The classical model of wound healing is divided into three sequential, yet overlapping phases, inflammatory, proliferate and remodeling [31]. Immediately after an injury, a fibrin clot is formed to limit the active bleeding (homeostasis). In the inflammatory phase, bacteria and debris are removed by phagocytic action. Proliferate phase is characterized by angiogenesis, collagen deposition, granulation tissue formation, epithelialization, and wound contraction [32]. Later part of the wound healing process involves remodeling the dermal tissues to produce greater tensile strength. The principal cell type involved in this process is the fibroblast [31].

Investigation of wound healing active constituents have been carried out employing both in-vivo methods and human trials in earlier times whereas at present, in-vitro methods based on cell cultures of fibroblasts, keratinocytes, epithelial cells and endothelial cells are used to identify the wound healing active plant secondary metabolites in addition to in-vivo methods [8]. The scratch wound assay has been established as a simple but valuable and inexpensive tool to obtain first insights into how plant preparations or their secondary metabolites can persuade formation of new tissue [33]. Scratch wound assay is particularly suitable for studying unidirectional cell migration and its regulation by intercellular interactions and interactions between cells with the extracellular matrix (ECM) in comparison with other popular in-vitro methods, such as time-lapse microscopy and Boyden chamber assays. During this wound healing assay, scratched cell monolayer responds to the disturbance of cell-cell contacts by increasing the concentration of growth factors and cytokines at the wound edge [34].

**Fig. 1:**
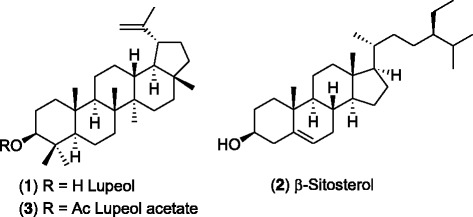
Chemical structures of lupeol, lupeol acetate and *β*-sitosterol

Herein we report the identification of lupeol (**1**) and *β*-sitosterol (**2**) (Fig. 1) as the potential wound healing active constituents from extracts of stem bark of *F. racemosa* and that lupeol acetate (**3**) acts as a prodrug for wound healing, through bioactivity directed fractionation using scratch wound assay (SWA) over Baby Hamster Kidney (BHK 21) and Madin-Darby Canine Kidney (MDCK) epithelial cell lines.^1^ It is known that keratinocytes, which produce keratin, are one of the main cell types that initiate cell proliferation and cell migration leading to closure of a wound. MDCK cells are reported to be capable of producing keratin [35]. Hence, although MDCK cell line is a general model of epithelial cells it has been used as an in-vitro model in wound healing assay [36].

## Methods

### Materials

Solvents used for the chemical extraction were of either analytical grade or general purpose reagent grade purified by fractional distillation. Chemicals and regents were purchased from Merck, Sigma-Aldrich, Fluka, Himedia and Invitrogen. Silica gel 60 G (Fluka − 60741) was used as solid phase, for gravity columns. Analytical and preparative Thin Layer Chromatography (TLC) was performed on pre-coated 0.25 mm thick plates of silica gel 60 F_254_ (Merck) or on glass plates (20 cm × 20 cm) coated with a mixture of silica gel 60 G (Fluka 60760) and silica gel 60 GF_254_ (Fluka 60765), 0.1 & 0.25 mm thickness. Prior to elution, plates were oven dried 80 °C to remove any adsorbed water. For visualization of TLC plates, UV-illumination (254 nm, 365 nm) and anisaldehyde were used. Analytical HPLC was performed on an Agilent Technologies, 1260 Infinity HPLC System equipped with an Agilent 1260 Infinity Series Diode Array Detector VL+ and utilizing OpenLAB CDS ChemStation VL software. HPLC separations were carried out using InertSustain© RP-C18 column (5 μm, 4.6 × 150 mm). Melting points were recorded using KSP 11 KPUSS A Kross optronic (Germany) melting point apparatus. 1D & 2D NMR spectra were recorded in CDCl_3_ with a Bruker Avance III 400 spectrometer at 400 MHz for ^1^H NMR and 100 MHz for ^13^C NMR using residual CHCl_3_ as the internal reference. Both BHK 21 and MDCK cell lines were purchased from American Type Culture Collection (ATCC®), Manassas, Virginia, USA.

### Scratch wound assay (SWA)

BHK 21(ATCC, CCL-10) and MDCK (ATCC, PTA 6502) cell cultures were established in the laboratory using standard in-vitro methods. Cells were grown in tissue culture flasks (25 cm^2^) in Dulbecco’s Modified Eagles Medium (DMEM) containing Fetal Bovine Serum supplemented with antibiotics [50 IU / mL penicillin and 50 μg/mL streptomycin (all Sigma-Aldrich–USA)]. Cultures were maintained at 37 °C in a 5% CO_2_ humidified incubator. Cell cultures were screened periodically for mycoplsama, bacterial and fungal contamination. A batch of seed samples in cryopreserving ampoule was stored in liquid nitrogen vessel for future use. Cells which grow at confluent stage were harvested from tissue culture vessel by centrifugation. Healthy cells were seeded in clear bottom 12 well plates in 10% DMEM growth medium at high density (3 × 10^4^ cells per well). Plates were incubated at 37 °C for 24 h in humidified incubator. Cultures were observed with an inverted microscope, equipped with digital camera for the formation of monolayer.

A scratch (wound) was performed on monolayer of cells along the vertical axis of each well under the microscope. The monolayer with wound was washed with 750 *µ*L of phosphate buffer saline (PBS). Each test well was filled with 990 *µ*L of DMEM and added 10 *µ*L of DMSO containing appropriate amount of test sample. Two negative controls, 1% DMSO in DMEM and 100% DMEM were used in this experiment. In addition, asiaticoside (25*µ*M), a potent wound healing agent [37] was used as the positive control when pure compounds were assayed. Plates were incubated for 24 h at 37 °C with 5% CO_2_. The widths of the wounds at different time intervals (0 h, 12 h, 18 h, and 24 h) were measured and the cell migration enhancement is presented as the percentage wound closure. Images at each stage were photographed. All the experiments were carried out in three replicates and three measurements were taken for each wound.

### Anti-bacterial assay: Kirby-Bauer disk diffusion method

#### Preparation of discs

All the extract and fractions were applied at a dose of 500 *µ*g / disc. Test samples were dissolved in MeOH (10 *µ*L) and adsorbed on to filter paper discs (Diameter = 6 mm). The negative control was prepared by adding MeOH (10 *µ*L) to a disc. Discs containing samples and control discs were dried in a vacuum oven maintained at 30 °C for 24 h in order to remove all traces of solvents. A disc containing 25 *µ*g of Amoxycillin (Himedia) were used as positive control [38].

#### Culture preparation

Antibacterial assay was carried out using against *Bacillus subtilis* (ATCC 6633), *Escherichia coli* (ATCC 25922), *Staphylococcus aureus* (ATCC 25923) and *Pseudomonas aeruginosa* (ATCC 27853) (Bacterial culture collection, Department of Botany, The Open University of Sri Lanka) in the preliminary investigation and the fractionation was selectively guided by the antibacterial assay against most effective pathogens. Bacterial species were grown as pure cultures on nutrient agar (NA) (Himedia-M001) plates and incubated for 24 h at 35 °C. Each culture was made into a suspension in sterilized distilled water up to a certain density, which was measured as a predetermined optical density (OD value); 0.05 OD at λ = 500 nm for *Bacillus subtilis*, 0.1 OD at λ = 500 nm for *E. coli*, *S. aureus* and *P. aeruginosa*. From each suspension 0.1 mL aliquots were transferred on to different NA plates and were spread using a sterilized spreader to prepare a uniform lawn of each bacterial species under study. The positive control, negative control and sample discs were placed on each plate. All experiments were done twice in duplicate and plates were incubated at 35 °C for 24 h. The zone of inhibition if present was measured and the mean with the standard errors were calculated.

### Anti-fungal assay: Kirby-Bauer disc diffusion method

#### Preparation of discs

All the extract and fractions were applied dose of 500 *µ*g / disc. Test samples were dissolve in MeOH (10 *µ*L) and adsorbed in to filter paper discs. The negative control was prepared by adding MeOH (10 *µ*L) to discs. All the discs were dried in a vacuum oven maintained at 30 °C for 24 h in order to remove trace of solvents. Disc containing 300 *µ*g of Polymyxin B (Himedia) were used as positive control [38].

#### Culture preparation

Antifungal assay was carried out against two S*accharomyces cervisiae* (NCYC 2401, NCYC 2402), a locally isolated S*accharomyces* sp. (Fungal culture collection of the Department of Botany, Sri Jayewardenepura University, Sri Lanka) and *Candida albicans* (ATCC 10231). Pure cultures of above fungi were grown as pure cultures on Yeast Malt Agar (YMA) (Himedia-M) plates and incubated for 24 h at 35 °C. Suspensions of these cultures were prepared in sterile distilled water having predetermined cell densities measured as OD value: 0.05 OD at λ = 500 nm for *Saccharomyces sp.,* 0.1 OD at λ = 500 nm for *Candida albicans*. A uniform lawn of each fungal species was prepared by spreading 0.1 mL aliquots from each suspension on different YMA plates. The positive control negative control and sample discs were placed on each plate. All experiments were done twice in duplicate and plates were incubated at 35 °C for 24 h. The zones of inhibition if present were measured and the mean with the standard errors were calculated.

### Extraction of the plant material

Dried powdered bark of *F. racemosa* L. was provided by Link Natural Products (Pvt) Ltd. Sri Lanka, in May 2011. Plant specimens were identified by Mr. T. M. S. G. Tennakoon, Head R & D section, Link Natural Product Ltd., Sri Lanka comparing *F. racemosa* L. specimens deposited in the Herbarium at Peradeniya Royal Botanical gardens, Sri Lanka and consulting the relevant literature [39]. A voucher specimen (68–002-01) was deposited at the herbarium of Link Natural Products Ltd. Sri Lanka. Plant material (500 g) was sequentially extracted with hexanes (9.32 g, 1.864%), dichloromethane (CH_2_Cl_2_) (1.35 g, 0.264%), ethyl acetate (EtOAc) (1.25 g, 0.250%), and methanol (MeOH) (1.21 g, 0.242%), in a Soxhlet apparatus and the solvents were removed under vacuum at 40 °C to obtain the respective extracts.

### Bioassay guided fractionation

The hexanes and CH_2_Cl_2_ extracts which showed cell migration enhancement activity was subjected to further fractionation using silica gel column chromatography. Column fractions were subjected to SWA at a concentration of 50 mg dm^− 3^. The percentage wound closure was calculated at 24 h. Further fractionation of the cell migration enhancement active fractions was carried out and the fractions were subjected to SWA at 25 mg dm^− 3^ and the fractions which showed wound healing activity were subjected to further fractionation to isolate potential cell migration enhancing compounds.

### Fractionation of the CH_2_Cl_2_ extract

The CH_2_Cl_2_ extract (1.0 g) of *F. racemosa* bark was chromatographed over a column of silica gel (30.0 g) made up in hexanes and eluted with hexanes containing increasing amounts of CH_2_Cl_2_, CH_2_Cl_2_ and CH_2_Cl_2_ containing increasing amounts of MeOH and finally washed with MeOH yielding 77 fractions. The column fractions were combined according their TLC patterns to give 18 major fractions (DF_1_–DF_18_) and were subjected to scratch wound assay. Column fractions which showed > 65% cell migration enhancement activity were further fractionated to isolate the active constituents using column and preparative thin layer chromatography.

### Fractionation of the hexanes extract

The hexane extract (2.5 g) of *F. racemosa* bark was chromatographed over a column silica gel (60 g) made up in hexanes and eluted with hexanes, hexanes containing increasing amounts of CH_2_Cl_2_, CH_2_Cl_2_ and CH_2_Cl_2_ containing increasing amounts of MeOH and finally washed with MeOH yielding 52 fractions. These column fractions were combined according their TLC patterns to give 11 major fractions (HF_1_–HF_11_) and were subjected to scrath wound assay. Column fractions which showed > 65% wound healing activity were further fractionated to isolate the active constituents using column and preparative thin layer chromatography.

### Conversion of lupeol (1) into lupeol acetate (3)

A sample of lupeol (2.5 mg) was mixed with pyridine (0.75 mL) and acetic anhydride (0.05 mL) in a round bottom flask and stirred for 6 h (progress of the reaction was monitored by TLC). The reaction mixture was neutralized with dilute HCl and extracted with ether, dried over anhydrous Na_2_SO_4_ and evaporated to dryness at 40 °C. The product was purified using preparative TLC (eluent: hexanes: CH_2_Cl_2_ 6:4 × 2) to obtain lupeol acetate (**3**) (1.7 mg; 68%; m. p. 217 °C).

### Statistical analysis

All the results are presented in this paper as mean ± standard error (*n* = 3 for SWA and *n* = 4 for antimicrobial assay). Mean comparisons, were performed using ANOVA/LSD post hoc test. *P*-values of less than 0.05 were considered to be significant. All statistical tests were carried out using the computer programme SPSS Version 20.0 computer programme.

## Results

### Evaluation of the wound healing effect of extracts and fractions of stem bark of *F. racemosa*

**Fig. 2:**
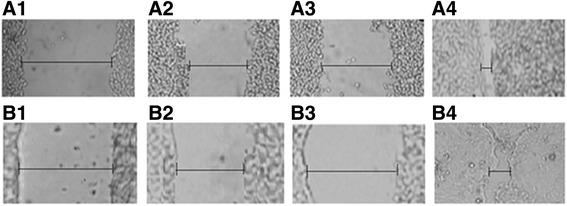
Images of the scratch wound assay of dichloromethane extract of *F. racemosa* on (a) BHK cells, A1- control at *t* = 0 h, A2- control at *t* = 24 h, A3- DCM extract at *t* = 0 h, A4- DCM extract at *t* = 24 h; (b) MDCK cells, B1- control at *t* = 0 h, B2- control at *t* = 24 h, B3- DCM extract at *t* = 0 h, B4- DCM extract at *t* = 24 h

Results indicated that the hexanes and dichloromethane extracts of the stem bark of *F. racemosa* has statistically significant effect on enhancing the wound healing (*p* ≤ 0.05) and the percentage of wound closure has been increased over both tested cell lines at 24 h (Table 1). Images of the SWA upon MDCK and BHK cells using the DCM extract are shown in Fig. 2.

**Table 1:** Weights of different extracts of *F. racemosa* bark and SWA results of those extracts with two different cell lines

Sample^a^	Weight of extract (g)	% Closure of the wound at *t* = 24 h^b^	
		BHK	MDCK
Hexane extract	9.32	91.0 (0.4)	84.9 (0.1)
CH_2_Cl_2_ extract	1.35	78.1 (0.9)	75.8 (0.1)
EtOAc extract	1.25	8.8 (0.8)	6.5 (0.1)
MeOH extract	1.21	14.3 (2.6)	7.4 (0.1)
1% DMSO (Control 1)		15.5 (1.2)	4.4 (0.9)
100% DMEM (Control 2)		16.5 (1.1)	4.9 (0.1)

^a^Sample concentration at a 100 mg dm^− 3^

^b^The mean value followed by the standard error of the mean within the parentheses

### Fractionation of the CH_2_Cl_2_ (DCM) extract and isolation and identification of wound healing active constituents

**Fig. 3:**
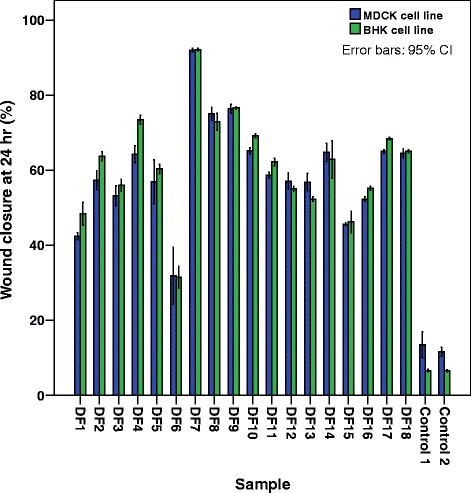
Percentage wound closure in the presence of column fractions of *F. racemosa* DCM extract on BHK and MDCK cell lines. (Bars represent the mean and confidence intervals of 95% of nine measurements in the three experiments)

Column chromatographic separation of the DCM extract (1.0 g) of *F. racemosa* bark yielded 18 major fractions [DF_1_ (10.6 mg), DF_2_ (10.8 mg), DF_3_ (39.3 mg), DF_4_ (5.2 mg), DF_5_ (2.8 mg), DF_6_ (3.7 mg), DF_7_ (21.7 mg), DF_8_ (20.9 mg), DF_9_ (39 .0 mg), DF_10_ (3.5 mg), DF_11_ (45.0 mg), DF_12_ (28.0 mg), DF_13_ (501.3 mg), DF_14_ (75.0 mg), and DF_15_ (60.0 mg), DF_16_ (55.2 mg), DF_17_ (91.1 mg), DF_18_ (50.7 mg)]. These fractions were subjected to SWA at a concentration of 50 mg dm^− 3^ and the fraction DF_7_ was found to be the most active fraction (Fig. 3). Further fractionation of fraction DF_7_ (4.0 mg) by preparative TLC (20 cm × 10 cm: 1 plate eluent: CH_2_Cl_2_) led to the isolation of the wound healing active compound which was identified as lupeol (**1**) (2.9 mg; 0.004%) by spectroscopic data and comparison with an authentic sample, m. p. 213–215 °C) (lit. 215 °C) [40] by spectroscopic data and comparison with an authentic sample (TLC, Co-TLC and mixed m. p.). ^1^H NMR (CDCl_3_, 400 MHz): *δ*_ppm_ 4.71, 4.59 (2H, *s*, H-29a, 29b), 3.21 (1H, *m*, H-3), 0.79, 0.81, 0.85, 0.97, 0.99, 1.05, 1.38 (each 3H, *s*). ^13^C NMR (CDCl_3_, 100 MHz): *δ*_ppm_ 151.0(C-20), 109.2(C-29), 79.4(C-3), 55.3(C-5), 50.4(C-9), 48.3(C-18), 47.9(C-19), 43.0(C-17), 42.9(C-14), 40.9(C-8), 40.0(C-22), 38.7(C-4), 38.6(C-13), 38.0(C-1), 37.2(C-10), 35.6(C-16), 34.3(C-7), 29.8(C-21), 28.0(C-23), 27.4(C-15), 27.1(C-12), 25.2(C-2), 20.9(C-11), 19.3(C-30), 18.3(C-6), 18.0(C-28), 16.1(C-25), 15.3(C-26), 14.7(C-24), 14.5(C-27).

TLC comparison of fractions DF_4_, DF_8_, DF_9_–DF_12_ with lupeol (**1**) indicated that lupeol is present in fractions DF_4_ and DF_8_ while fractions DF_9_–DF_12_ did not contain detectable amounts of lupeol. Fraction DF_9_ (35.0 mg) was further fractionated by column chromatography over a column of silica gel (1.0 g) made up in hexanes/CH_2_Cl_2_ (75:25) and eluted with hexanes/CH_2_Cl_2_ (65:35), hexanes/CH_2_Cl_2_ (50:50), hexanes/CH_2_Cl_2_ (25:75) and CH_2_Cl_2_ to give 4 major sub fractions [DF_9_1 (7.7 mg), DF_9_2 (3.9 mg), DF_9_3 (7.1 mg), DF_9_4 (6.6 mg)]. These fractions were subjected to SWA at a concentration of 25 mg dm^− 3^ and fractions DF_9_2 and DF_9_3 were found to be active. Fraction DF_9_3 was found to be constituted of (90%) a single compound and purification of this fraction (4 mg) on preparative TLC (20 cm × 10 cm: 1 plate eluent: CH_2_Cl_2_) led to the isolation of *β*-sitosterol (**2**) (3.4 mg; 0.002%) which was identified by spectroscopic data and comparison with an authentic sample. ^1^H NMR (CDCl_3_, 400 MHz): *δ*_ppm_ 5.33 (1H, m, H-6), 3.50 (1H, m, H-3), 1.02 (3H, s, H-29), 0.93 (3H, d, H-19), 0.85 (3H, t, H-24), 0.81 (3H, d, H-26), 0.69 (3H, s, H-27). ^13^C NMR (CDCl_3_, 100 MHz): *δ*_ppm_ 140.9 (C-5), 121.5 (C-6), 72.0 (C-3), 56.9(C-14), 56.2 (C-17), 50.3 (C-9), 46.0 (C-25), 42.5 (C-4, C-13), 39.9 (C-12), 37.5 (C-1), 36.7 (C-10), 36.3 (C-18), 34.1 (C-20), 32.1 (C-8), 31.8 (C-7), 31.7 (C2), 29.3 (C-23), 28.5 (C-16), 26.2 (C-21), 24.5 (C-15), 23.2 (C-22), 21.3 (C-11), 20.0 (C-26), 19.6 (C-27), 19.2 (C-19), 18.9 (C-28), 12.2 (C-24),12.0 (C-29). Fractions DF_9_2 and DF_10_–DF_12_ also showed the presence of *β*-sitosterol (**2**) by TLC.

### Fractionation of the hexanes extract and isolation and identification of wound healing active constituents

**Fig. 4:**
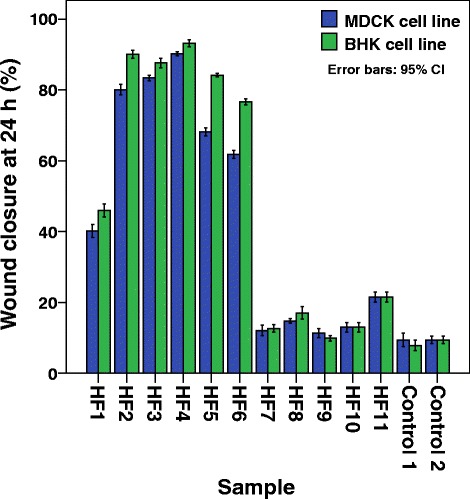
Percentage wound closure in the presence of column fractions of *F. racemosa* hexanes extract on BHK and MDCK cell lines. (Bars represent the mean and confidence intervals of 95% of nine measurements in the three experiments)

Column chromatographic separation of the Hexanes extract (2.5 g) of *F. racemosa* bark yielded 11 major fractions, [HF_1_ (233.8 mg), HF_2_ (1314.8 mg), HF_3_ (19.7 mg), HF_4_ (5.1 mg), HF_5_ (20.5 mg), HF_6_ (8.8 mg), HF_7_ (103.6 mg), HF_8_ (157.7 mg), HF_9_ (94.5 mg), HF_10_ (289.3 mg), HF_11_ (31.5 mg)]. These fractions were subjected to SWA assay at a concentration of 50 mg dm^− 3^ and the fraction HF_4_ was found to be the most active fraction (> 90%) while fractions HF_2_, HF_3_, HF_5_ and HF_6_ also showed enhanced (> 65%) wound healing activity (Fig. 4). The fraction HF_4_ (5.0 mg) was further fractionated using preparative TLC (20 cm × 20 cm, 1 plate, eluent: 20% hexanes in CH_2_Cl_2_) to obtain five fractions (HF_4_1–HF_4_5) (Table 2). Sub fraction HF_4_2 showed significant wound healing activity (*p* ≤ 0.05) at a concentration of 25 mg dm^− 3^ against both cell lines (Table 2). It was found to contain a single compound and was identified as lupeol (**1**) (2.2 mg; 0.002%).

**Table 2:** Weights of sub fractions of fraction HF_4_ of hexanes extract and percentage wound closure of those fractions at 24 h with two different cell lines

Sample^a^	Weight of fraction (mg)	% Closure of the wound at *t* = 24 h^b^	
		BHK	MDCK
HF_4_	–	85.1 (0.2)	83.9 (0.2)
HF_4_ 1	1.1	27.9 (0.1)	24.1 (0.2)
HF_4_ 2	2.2	83.6 (0.2)	80.0 (0.1)
HF_4_ 3	0.9	58.6 (0.3)	55.0 (0.2)
HF_4_ 4	0.2	55.1 (0.2)	55.3 (0.2)
HF_4_ 5	0.2	24.1 (0.2)	22.2 (0.1)
1% DMSO (Control 1)		10.6 (0.3)	10.0 (0.4)
100% DMEM (Control 2)		13.5 (0.5)	11.1 (0.5)

^a^Sample concentration at a 25 mg dm^−3^

^b^The mean value followed by the standard error of the mean within the parentheses

Fraction HF_2_ (500 mg) was chromatographed over a column of silica gel to give 13 major sub fractions [HF_2_1 (2.0 mg), HF_2_2 (25.0 mg), HF_2_3 (265.0 mg), HF_2_4 (26.0 mg) HF_2_5 (11.0 mg), HF_2_6 (32.0 mg), HF_2_7 (7.0 mg), HF_2_8 (9.0 mg) HF_2_9 (11.0 mg), HF_2_10 (4.0 mg), HF_2_11 (4.0 mg), HF_2_12 (51.0 mg) HF_2_13 (15.0 mg)]. The sub fractions HF_2_9–HF_2_13 showed a considerable cell migration enhancement (55%–75%) at 24 h all of which contained varying amounts of lupeol. Although the sub fraction HF_2_6 did not show a considerable cell migration enhancement at 24 h, it has shown a substantial cell migration enhancement at 48 h over both cell lines. Fraction HF_2_6 constituted of one major compound. Purification of this fraction (30 mg), using preparative TLC (eluent: hexanes: CH_2_Cl_2_ 6:4 × 2) yielded a white crystalline compound (29 mg; 0.060%), which was identified as lupeol acetate (**3**) by TLC, Co-TLC. Its identity was confirmed by m. p. (216–217 °C) (lit. 218 °C) [40] mixed m. p. with an authentic sample and chemical conversion of lupeol into its acetate.

**Fig. 5:**
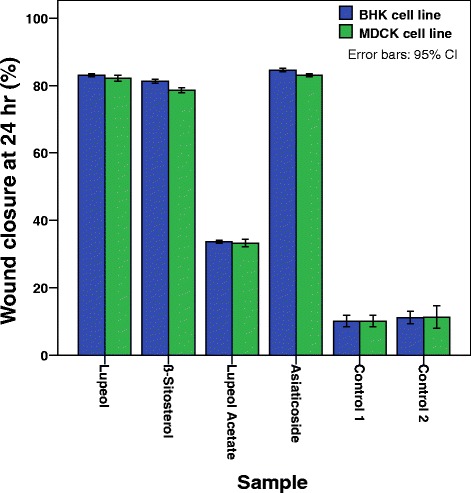
Percentage wound closure in the presence of lupeol (**1**), β-sitosterol (**2**), lupeol acetate (**3**) and positive control asiaticoside

All three pure compounds, lupeol (**1**), *β*-sitosterol (**2**), and lupeol acetate (**3**) were subjected to SWA at 25 *µ*M concentration along with asiaticoside as the positive control and the percentage closure of wound at 24 h was obtained (Fig. 5).

**Fig. 6:**
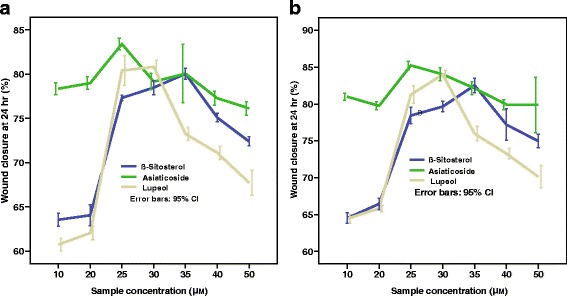
Variation of percentage wound closure with concentration in the presence of lupeol (**1**), β-sitosterol (**2**) and asiaticoside (positive control) at 24 h: (**a**) BHK cell line, (**b**) MDCK cell line. (Bars represent the mean and confidence intervals of 95% of nine measurements in the three experiments)

Optimum concentrations of lupeol (**1**) and *β*-sitosterol (**2**) required for the maximum cell migration enhancement activity at 24 h were determined by carrying out the SWA for these two compounds with asiaticoside as the positive control at different concentrations (10–50 *µ*M) and the results are shown in Fig. 6. It was found that optimal concentrations of 1, 2, and positive control are 30 *µ*M, 35 *µ*M and 25 *µ*M respectively against both cell lines.

### Role of lupeol acetate (**3**) in wound healing

**Fig. 7:**
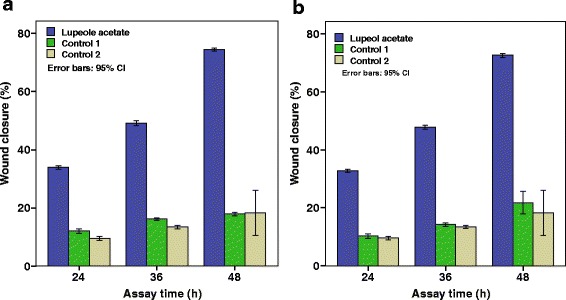
Percentage wound closure in the presence of lupeol acetate (**3**) with time. **a** BHK cell line. **b** MDCK cell line. (Bars represent the mean confidence intervals of 95% of nine measurements in the three experiments)

**Fig. 8:**
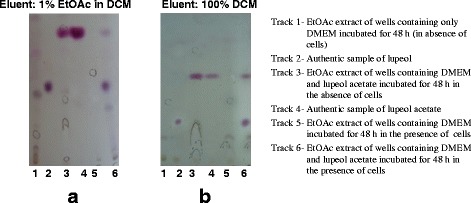
Thin Layer Chromatographic comparison of the EtOAc extracts showing hydrolysis of lupeol acetate during scratch wound assay (SWA) (**a**) BHK Cells. (**b**) MDCK cells. Spray reagent: Anisaldehyde spray reagent

Although lupeol acetate (**3**) has not shown a considerable cell migration enhancement activity in 24 h, it was observed that the cell migration enhancement activity increases with time and showed percentage healing of 74% in BHK cell line and 72% in MDCK cell line at 48 h (Fig. 7). This observation prompted us to investigate whether lupeol acetate acting as a pro-drug and undergo hydrolysis in the vicinity of the wound to produce lupeol (**1**). In order to test this hypothesis, we incubated lupeol acetate in DMEM (10 mg dm^− 3^) in the presence of cells with a wound and absence of cells and tested the medium for the presence of lupeol (by TLC) with time while simultaneously monitoring the cell migration enhancement activity. Comparative TLC examination of the two assay media revealed that, lupeol acetate (**3**) has been hydrolyzed to give lupeol (**1**) in the presence of the cells while no change has occurred in the absence of cells (Fig. 8), revealing that lupeol acetate act as a pro-drug in the vicinity of cells.

### Anti-bacterial activity of extracts of *F. racemosa*

**Fig. 9:**
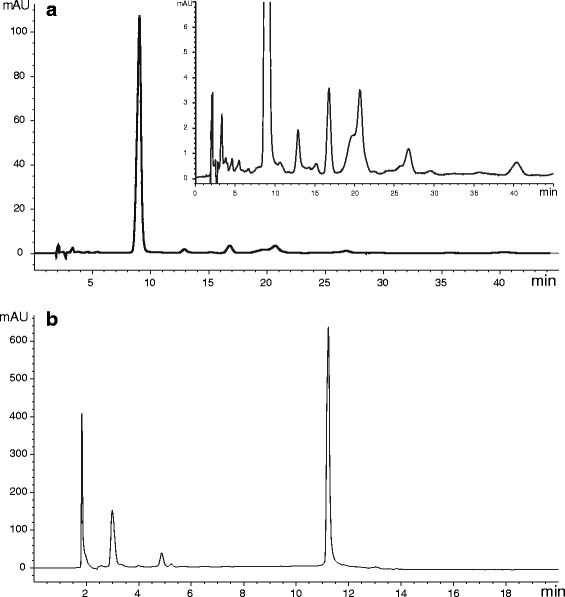
HPLC traces of (**a**) EtOAc extract (insert shows the same HPLC trace with the expansion of Y axis) (isocratic elution with 90:10 MeCN− 1.0% HOAc in H_2_O at a rate of 1 mL/min at 30 °C) and (**b**) MeOH extract of *F. racemosa* (stepwise gradient elution with MeCN− 1.0% HOAc in H_2_O as follows; 0.00–4.00 min with 10:90, 4.00–8.00 min with 15:85, 8.00–10.00 min with 20:80 and 10.00–20.00 with 100% MeCN)

**Fig. 10:**
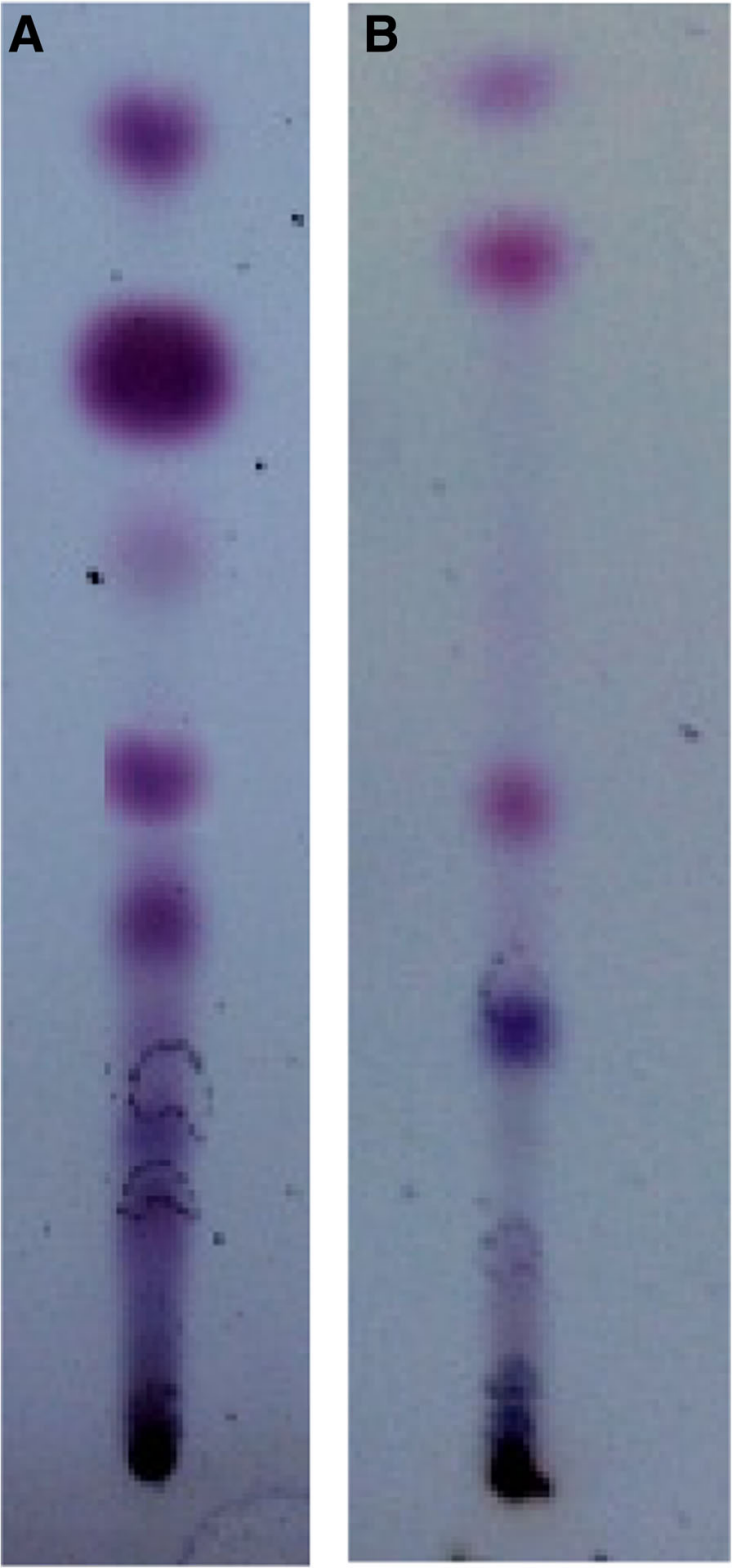
TLC profiles of (**a**) hexane extract (eluent: 20% CH_2_Cl_2_ in hexanes) and (**b**) CH_2_Cl_2_ extract (eluent: CH_2_Cl_2_) of *F. racemosa*

It was found that the EtOAc and the MeOH extracts were active against the two gram positive bacteria *B. subtilis* and *S. aureus*, at a dose of 500 *µ*g/disc but inactive against the gram negative bacteria species *E. coli* and *P. aeruginosa* (HPLC traces of EtOAc and MeOH extracts are shown in Fig. 9). The hexanes and CH_2_Cl_2_ extracts did not show activity against any of the tested organisms (TLC profiles of hexanes and CH_2_Cl_2_ extracts of *F. racemosa* are shown in Fig. 10). Disc containing Amoxicillin 25 *µ*g served as the positive control. Results obtained against the four different bacterial strains tested are tabulated in Table 3. However it was not possible to isolate the active constituents because they were inseparable on normal and reversed phase TLC.

**Table 3:** Mean diameters of clear zones developed with different extracts of *F. racemosa* with *Bacillus subtilis, Staphylococcus aureus, Pseudomonas aeruginosa* and Escherichia coli

	Diameter of clear zone (mm)*			
	*B. subtilis*	*S. aureus*	*P. aeruginosa*	*E. coli*
Hexane	–	–	–	–
CH_2_Cl_2_	–	–	–	–
EtOAc	10.44 (0.15)	10.50 (0.19)	–	–
MeOH	9.63 (0.16)	9.56 (0.24)	–	–
Positive control	12.13 (0.18)	11.44 (0.24)	9.63 (0.21)	12.13 (0.18)

*The mean value followed by the standard error of the mean within the parentheses

### Anti-fungal activity of extracts of *F. racemosa*

It was found that the EtOAc and the MeOH extracts were active against the all four fungi species at a dose of 500 *µ*g/disc (HPLC traces of EtOAc and MeOH extracts are shown in Fig. 9). The hexanes and CH_2_Cl_2_ extracts did not show activity against any of the tested organisms (TLC profiles of hexanes and CH_2_Cl_2_ extracts of *F. racemosa* are shown in Fig. 10). Disc containing Polymyxin B 300 *µ*g served as the positive control. Results obtained against three different *Saccharomyces* spp. and *Candida albicans* are tabulated in Table 4.

**Table 4:** Anti-fungal activity of different extracts of *F. racemosa* on *Saccharomyces* spp. and *Candida albicans*

Extract	Fungi / diameter of clear zone (mm)*			
	*S. cervisiae* NCYC 2401	*S. cervisiae* NCYC 2402	*Saccharomyces* sp.	*C. albicans*
Hexane	–	–	–	–
CH_2_Cl_2_	–	–	–	–
EtOAc	10.69 (0.23)	8.38 (0.21)	9.38 (0.23)	10.69 (0.19)
MeOH	10.38 (0.16)	7.31 (0.13)	8.94 (0.18)	9.94 (0.18)
Positive control	12.12 (0.18)	11.44 (0.24)	9.38 (0.23)	12.13 (0.18)

*The mean value followed by the standard error of the mean within the parentheses

## Discussion

In the treatment of wounds, shortening of the healing time and protecting the wound from bacterial infections are two main objectives. The open blood vessels and tissues in a wound area is a favorable place for multiplication of microbes because of provision of proper environment needed for efficient growth of microbes. As such, in addition to enhancing cell proliferation, prevention of microbial invasion of the wound is a paramount important requirement in the effort of wound healing [41]. The main objective of the antimicrobial therapy in wound care is to control microbial colonization and simultaneously increase cell proliferation, in order to enhance the wound healing [42]. It has been reported that herbal medicines have an array of beneficial properties such as efficacy, nontoxicity, availability, affordability as they are cheaper than the conventional synthetic drugs [43]. Some herbal extracts either directly or indirectly promote wound repair or exhibit antimicrobial properties there by prevent wound infestation. It is important to note that, the antimicrobial property of a drug also plays an important role in wound care. In our experimental work, the biological activities shown by the extracts of *F. racemosa* were found to be playing a dual role in the healing of wounds. According to the antimicrobial assay results both methanol and ethyl acetate extracts of stem bark of *F. racemosa* showed antimicrobial activity against gram positive bacteria in addition to the enhanced cell migration activity of hexanes and dichloromethane extracts of the same over both cell lines tested at the 24 h.

Based on the results of bioassay guided fractionation, it is revealed that lupeol (**1**) and *β*-sitosterol (**2**) are the cell migration enhancing compounds present in dichloromethane extract of the bark of *F. racemosa*. It was reported that lupeol (**1**) isolated from leaves of *Celastrus paniculaates* has shown potential wound healing activity on excision, incision and dead space wounds in albino rats [29] while *β*-sitosterol isolated from *Aloe vera* gel has shown a potential angiogenic activity in the chorioallantoic membrane of chick embryo and stimulates the migration of human umbilical vein endothelial cells [44]. It is also reported that lupeol possesses anti-inflammatory activity which too would have an impact on its wound healing activity [45].

It is also revealed, that, both lupeol (**1**) and *β*-sitosterol (**2**) exhibit a significant cell migration enhancement in both cell lines at 24 h which is comparable with that of the positive control, asiaticoside (Fig. 5). Both hexanes and dichloromethane extracts contain lupeol (**1**) while lupeol acetate (**3**) present only in hexanes extract and *β*-sitosterol (**2**) found only in the dichloromethane extract. The observed cell migration enhancement activity of **3** could be attributed to its hydrolysis into **1** in the vicinity of wound area. As lupeol acetate (**3**) enhances the cell migration with time by undergoing slow hydrolysis to give lupeol (**1**) in the presence of the cells while no hydrolysis of **3** occurred in the absence of cells (Fig. 6), it is revealed that lupeol acetate act as a pro-drug in the vicinity of cells. Lupeol acetate which co-exists with lupeol in the hexane extract may improve and assist the wound healing process with time. As different extracts of bark of *F. racemosa* exhibit both cell migration enhancing as well antimicrobial properties, it could be concluded that a drug developed from this plant extract may have dual action in wound healing.

## Conclusion

In the present study we have investigated the wound healing potential of *F. racemosa* bark extractives using SWA as an in-vitro wound healing model and were able to identify the possible cell migration enhancing constituents as lupeol and *β*-sitosterol via bio activity directed fractionation procedures. The cell migration enhancement activity of each of these two compounds is concentration dependent and exhibits an optimum value comparable to that of the positive control, asiaticoside. It is also found that lupeol acetate (**3**) which is present in *F. racemosa* bark hydrolyzes to form lupeol (**1**) in the presence of cells and thereby acts as a pro-drug in enhancing the cell migration. The ethyl acetate and methanol extracts of the plant exhibited anti-microbial activity against *Staphyllococus, Bacillus* and *Saccharomyces* species as well as *Candida albicans.* The combined anti-microbial effect and enhancement of cell migration effect may aid in wound healing, and our results support the use of *F. racemosa* L. in the traditional medicine for wound healing.

## Abbreviations

ATCC: American type culture collection
BHK: Baby hamster kidney
DMEM: Dubelco’s modified eagle media
DMSO: Dimethyl sulfoxide
ECM: Extracellular matrix
EtOAc: Ethyl acetate
FBS: Fetal bovine serum
HPLC: High performance liquid chromatography
MDCK: Madin-Darby canine kidney
MeOH: Methanol
NA: Nutrient agar
NCYC: National collection of yeast culture
NMR: Nuclear magnetic resonance spectroscopy
PBS: Phosphate buffer saline
SWA: Scratch wound assay
TLC: Thin layer chromatography
YMA: Yeast malt agar

## Footnotes

1 Part of this work was submitted to Institute of Chemistry, Ceylon by NSB for Kandiah Memorial Award for the best postgraduate research work in Basic Chemistry 2016. The award lecture has been published; Bopage NS. Investigation on Wound healing activity of bark of *Ficus racemosa* and “Seetodaka” oil using scratch wound assay (SWA). *Chemistry in Sri Lanka*. 2016; 33:18–21.
